# PD51 Strategies For Reducing Cesarean Section Rates: An Overview Of Organizational Interventions

**DOI:** 10.1017/S0266462325101839

**Published:** 2025-12-29

**Authors:** Avila Vidal, Jorge Barreto

## Abstract

**Introduction:**

The increase in Cesarean section (C-section) rates is a global phenomenon, but it is mainly observed in Latin America and the Caribbean, which from the 1990s through to 2014 saw the average rate of C-sections increase from 22.8 to 42.2 percent. Recommendations from international organizations on the need to reduce rates of C-sections, as well as initiatives in several countries, can be highlighted in this scenario.

**Methods:**

This study involved an overview of systematic reviews to evaluate interventions aimed at reducing rates of C-sections. The methodological quality of the reviews was assessed using the AMSTAR tool. Comprehensive searches were performed in indexed databases, including PubMed, the Cochrane Library, the Virtual Health Library Regional Portal, Epistemonikos, and Health Systems Evidence. Identified interventions were classified using the Effective Practice and Organization of Care (EPOC) taxonomy from the Cochrane Collaboration, ensuring a structured evaluation of effective practices and care organization.

**Results:**

Out of 969 identified studies, twelve were duplicates. Following the screening of titles and abstracts, nine studies were selected for full-text analysis, ultimately resulting in six studies included in the review. According to the EPOC taxonomy, the most prevalent intervention types were financing and mixed interventions. Notably, mixed interventions, which combined implementation strategies and governance, demonstrated the most significant effectiveness in reducing C-section rates.

**Conclusions:**

The effectiveness of interventions to reduce rates of C-sections varied based on context and evidence certainty. Public policies must account for the existing healthcare system and cultural norms, ensuring that they adapt to local contexts to effectively address resistance. Tailoring strategies to align with community values is essential for successful implementation and for achieving desired outcomes in reducing C-section rates.

